# Adolescent stress accelerates postpartum novelty recognition impairment in 5xFAD mice

**DOI:** 10.3389/fnins.2024.1366199

**Published:** 2024-05-15

**Authors:** Owen Leitzel, Jose Francis-Oliveira, Shaimaa M. Khedr, Lila Ariste, Stefanie Robel, Shin-ichi Kano, Andrew Arrant, Minae Niwa

**Affiliations:** ^1^Department of Psychiatry and Behavioral Neurobiology, University of Alabama at Birmingham, Birmingham, AL, United States; ^2^Department of Cell, Developmental and Integrative Biology, University of Alabama at Birmingham, Birmingham, AL, United States; ^3^Department of Biology, Adelphi University, Garden City, NY, United States; ^4^Department of Neurobiology, University of Alabama at Birmingham, Birmingham, AL, United States; ^5^Department of Neurology, University of Alabama at Birmingham, Birmingham, AL, United States; ^6^Department of Biomedical Engineering, University of Alabama at Birmingham, Birmingham, AL, United States

**Keywords:** adolescent stress, Alzheimer’s disease, acceleration, corticosterone, novelty recognition impairment

## Abstract

Pregnancy and the postpartum period induce physiological changes that can influence women’s cognitive functions. Alzheimer’s disease (AD) has a higher prevalence in women and is exacerbated by early life stress. In the present study, we found that late adolescent social isolation combined with the experience of pregnancy and delivery accelerates the onset of cognitive deficits in 5xFAD dams, particularly affecting their ability to recognize novelty. These cognitive deficits manifested as early as 16 weeks, earlier than the usual timeline for these mice, and were closely associated with increased levels of corticosterone, suggesting dysregulation of the hypothalamic–pituitary–adrenal (HPA) axis. Notably, the presence of *β*-amyloid plaques in brain regions associated with novelty recognition did not significantly contribute to these deficits. This highlights the potential role of stress and HPA axis dysregulation in the development of cognitive impairments related to AD, and underscores the need for further investigation.

## 1 Introduction

The neuroendocrine, molecular, and physiological adaptations that occur during pregnancy, childbirth, and the postpartum period correlate with behavioral changes in maternal mood, cognition, and stress control (Leuner and Sabihi, 2016). These adaptations reflect the intricate interplay between hormonal fluctuations and neural responses, shaping the maternal brain in preparation for caregiving responsibilities (Brunton and Russell, 2008; Bale and Epperson, 2015; Morrison et al., 2020). Nonetheless, despite this profound influence, women have historically been underrepresented in the field of biomedical research, with studies often overlooking the unique physiological and psychological challenges faced during the reproductive years (Shors, 2016; Galea et al., 2018).

Alzheimer’s disease (AD) and other neurodegenerative disease conditions lead to severe cognitive decline by disrupting neural circuits responsive to stress (Shors, 2016; Deems and Leuner, 2020). Psychosocial stress, including social isolation, along with stress experienced during critical periods such as pregnancy, childbirth, and the postpartum period, can exacerbate cognitive vulnerabilities. This may lead to an acceleration in the onset or progression of symptoms related to AD, including cognition decline (Justice, 2018; Lesuis et al., 2018). In the United States alone, approximately 6.9 million individuals aged 65 and over suffer from AD, with two-thirds being women (Barnes et al., 2005; Snyder et al., 2016; Deems and Leuner, 2020; Rajan et al., 2021). Furthermore, the disproportionate prevalence of AD among women underscores the need to understand how sex-specific factors, including hormonal fluctuations and stress experiences unique to the female lifespan, contribute to cognitive decline and disease susceptibility (Nebel et al., 2018).

Recent studies using animal models, such as transgenic mouse models of AD, have provided valuable insights into the role of stress in accelerating cognitive impairment. For instance, observations in mice with AD-related genetic mutations (AD mice; e.g., 5xFAD mice, APP/PS1 mice) have demonstrated that early life stress, including social isolation, can expedite cognitive decline (Dong et al., 2004; Jeong et al., 2006; Han et al., 2016, 2017; Nakai et al., 2021). These findings not only shed light on the pathophysiological mechanisms underlying AD but also highlight the broader relevance of stress-induced cognitive impairments.

We have previously developed a novel concept that subtle psychosocial stress in late adolescence can have long-lasting negative effects on subsequent brain maturation and behaviors. Specifically, a mild three-week social isolation during late adolescence in mice differs notably from longer-term isolation (e.g., exposure for several weeks at various life stages studied by numerous groups), as it does not itself produce significant endocrine, neuronal, or behavioral abnormalities (Niwa et al., 2013, 2016; Matsumoto et al., 2017; Lockhart et al., 2018; Hikida et al., 2020; Kin et al., 2021). Expanding this paradigm further, we recently found that psychosocial stress, specifically social isolation during late adolescence, leads to deficits in postpartum social novelty recognition only in stressed dams, which are mice that experienced both social isolation and pregnancy/delivery (Kin et al., 2023; Niwa et al., 2024). Additionally, our recent study has identified glucocorticoid-mediated functional alteration of the anterior insula (AI) – prelimbic cortex (PrL) projections as an underlying cause of social novelty recognition deficits in stressed dams (Kin et al., 2023). Notably, pathological alterations in human AI, dorsolateral anterior cingulate cortex (homologous to the PrL in rodents), and dentate gyrus of hippocampus (DG) have been implicated in AD (Bonthius et al., 2005; Eimer and Vassar, 2013; Le Heron et al., 2018). Therefore, it is possible that genetic variants associated with AD, psychological stress before pregnancy, and stressful events later in life such as pregnancy and delivery, may interact to strongly accelerate AD-associated symptoms later in life. However, the biological mechanisms underlying the trajectory from psychological stress before pregnancy to the acceleration of AD-related novelty recognition impairment later in life remain to be elucidated. Thus, in the present study, we adapted this model to examine the effects of late adolescent social isolation on the acceleration of deficits in novelty recognition behaviors in 5xFAD dams and virgin female mice.

## 2 Materials and methods

### 2.1 Animals

B6SJL-Tg(APPSwFlLon,PSEN1*M146L*L286V)6799Vas/Mmjax (5xFAD) female mice were originally purchased from the Jackson Laboratory (#034840-JAX). All mice used in this study were bred in-house over 10 generations of 5xFAD mice. The 5xFAD mice express the human mutant amyloid precursor protein (APP) and presenilin 1 (PSEN1) transgenes, with three mutations in APP and two in PSEN1. These mice develop amyloid-β (Aβ) deposition and gliosis around 2 months of age, and by 5 months of age, they show synaptic loss and cognitive decline (Oakley et al., 2006). Previous studies highlight social behavioral deficits emerging around 9–12 months of age in these mice, while impairments in non-social cognition manifest as early as 5–6 months (Filali et al., 2011; Giannoni et al., 2013; Flanigan et al., 2014; Huang et al., 2016; Creighton et al., 2019; Kosel et al., 2019; Samaey et al., 2019). Given these robust pathological and behavioral features, the 5xFAD model was specifically chosen for the present study.

**Figure 1:**
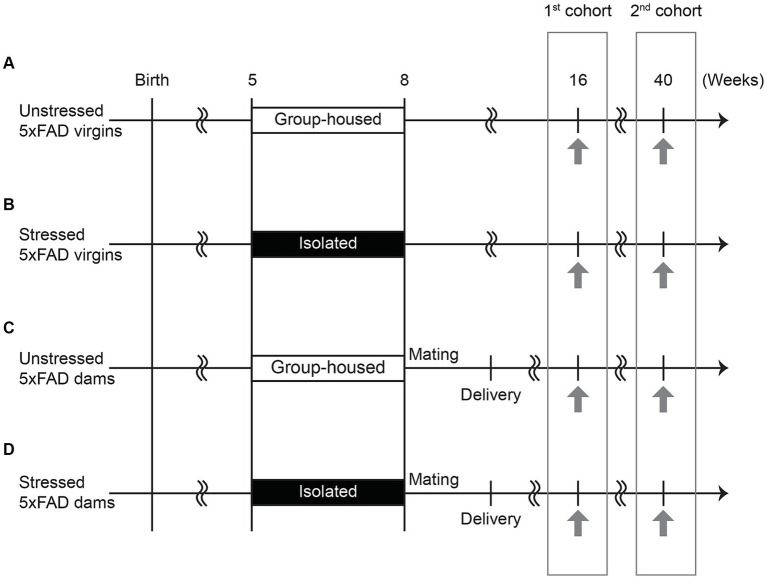
Experimental schedule. **(A)** Virgin 5xFAD females were group-housed and not mated (unstressed virgins). **(B)** Virgin 5xFAD females were isolated from 5 to 8 weeks of age and not mated (stressed virgins). **(C)** Virgin 5xFAD females were group-housed, mated with a male at 8 weeks of age, and gave birth to pups (unstressed dams). **(D)** Virgin 5xFAD females were isolated from 5 to 8 weeks of age, mated with a male at 8 weeks of age, and gave birth to pups (stressed dams). The novel object recognition test, followed by three-chamber social interaction test, was performed at 16 and 40 weeks of age (indicated by the arrows). Different cohorts of mice subjected to behavioral tests at 16 weeks and 40 weeks of age were prepared to avoid the repeated exposure to stressful behavioral procedures.

Mice were kept under standard conditions (23 ± 3°C; 40 ± 5% humidity; lights on at 6 am and off at 6 pm) with water and food *ad libitum*. Healthy virgin 5xFAD mice were group-housed or isolated from 5–8 weeks of age, corresponding to late adolescence — a critical period characterized by significant hormonal changes and the fine-tuning of neuronal pathways to facilitate the transition from childhood to adulthood (Blakemore, 2008). 5xFAD female mice were also either mated in-house with healthy C57BL/6 J male mice at 8 weeks of age, followed by pregnancy and delivery, or remained unmated. This design generated four experimental groups: unstressed virgins, stressed virgins, unstressed dams, and stressed dams. ‘Virgins’ denote nulliparous females, indicating mice with no prior mating, pregnancy, or delivery experience, while ‘dams’ denote primiparous females, indicating those that have given birth once. The experimental design and schedule are shown in Figure 1.

### 2.2 Behavioral tests

We performed two behavioral tests across multiple days, maintaining consistent conditions in the behavioral room throughout the testing period. The novel object recognition test (NORT) and social interaction test (SIT) were conducted at two specific time points: 40 weeks, a period when cognitive impairment is typically observed in 5xFAD mice, and 16 weeks, an earlier timeframe than the typical onset of such behavioral deficits (Giannoni et al., 2013; Creighton et al., 2019; Yanai and Endo, 2021). Supplementary Figure S1 illustrates a flowchart outlining our behavioral testing protocols and sample collection processes.

#### 2.2.1 Novel object recognition test (NORT)

Novel object recognition test was conducted with one cohort assessed at 16 weeks of age and a second cohort evaluated at 40 weeks of age, with minor modifications (Sun et al., 2020; Lee et al., 2021). Each trial consisted of three phases: habituation, training, and testing, each lasting 10 min. During the habituation phase, the subject mouse was introduced into a chamber apparatus (40 cm × 40 cm × 42 cm) and allowed to habituate for 10 min per day over the course of 3 days. On the fourth day, during the training phase, the mouse was once again habituated for 10 min in the chamber apparatus. Following this, the mouse was briefly removed and then reintroduced into the chamber, which contained two identical cone objects, for 2 min. Data during the training phase were collected over the subsequent 10 min. On the fifth day, the test phase began with a 10-min habituation in the chamber apparatus. Subsequently, the mouse was briefly removed from the chamber. During this brief removal, one of the two familiar cone objects was replaced with a novel pyramid object. The mouse was then returned to the chamber for 2 min, after which data collection took place over the following 10 min. All data were manually analyzed by reviewing video recordings to identify instances of nose contact or physical interactions within a 1 cm boundary zone around the objects. Stopwatch timers were used to measure the time spent sniffing. The discrimination index was calculated as follows: (time sniffing the novel object – time sniffing the familiar object)/(time sniffing the novel object + time sniffing the familiar object). An index of 0 indicated no recognition of the novel object, positive values indicated increased recognition of the novel object, and negative values indicated deficits in recognizing the novel object.

#### 2.2.2 Social interaction test (SIT)

Social interaction test was conducted as previously described with minor modifications (Moy et al., 2004; Kaidanovich-Beilin et al., 2011; Sandi and Haller, 2015; Kin et al., 2023; Niwa et al., 2024,). SIT consisted of two trials: the Sociability Trial (S-trial) and the Social Novelty Trial (SN-trial). These trials were designed to progressively increase the complexity of the social context. One day following the testing phase of NORT, the subject mouse was placed in the middle of a chamber apparatus (40 cm × 40 cm × 42 cm), which was divided into three chambers: left, middle, and right. The left and right chambers each contained small, barred cages. The subject mouse was allowed to habituate to the three-chamber environment for 10 min daily over three consecutive days before testing. After this habituation period, the mouse was placed into the middle chamber and allowed to habituate for 10 min before being confined to the middle chamber. The doors were then closed to create three separate chambers, and the subject mouse returned to the middle chamber for 5 min. During this period, a novel mouse was introduced into one of the barred cages, while a mouse-shaped object was placed in the other barred cage. In the S-trial, the subject mouse encountered the mouse in the barred cage and the object in the other barred cage for 10 min. At the 10-min mark, the test mice were again restricted from entering the right and left chambers for 5 min, and the mouse-shaped object was replaced with another novel mouse. During the SN-trial, the subject mouse encountered a familiar mouse in one barred cage and a novel mouse in the other barred cage for 10 min. Familiar and novel mice were chosen to be age- and sex-matched healthy mice. All data were manually analyzed by reviewing video recordings to identify instances of nose contact or physical interactions within a 1 cm boundary zone around the barred cages either containing mice or mouse-shaped objects. Stopwatch timers were used to measure the time spent sniffing. Sociability and social novelty indexes were calculated as follows: (time interacting with mouse or novel mouse cage – time interacting with an object or familiar mouse cage)/(time interacting with a mouse or novel mouse cage + time interacting with an object or familiar mouse cage). An index of 0 indicated no preference, while positive indexes indicated increased sociability or social novelty behavior, and negative indexes indicated social avoidance or deficits in social novelty behavior.

### 2.3 Blood collection and brain perfusion

Brain perfusion and blood collection were performed with minor modifications to the methods previously described in the publications (Niwa et al., 2013, 2024). To minimize potential circadian rhythm influences and the acute effects of stressful behavioral testing, blood collection consistently occurred at the same time of day, 24 h after the last behavioral testing session. Specifically, 1 day following the SN-trials for both the 16 and 40 weeks of age groups, mice were anesthetized with isoflurane. Subsequently, 1 mL of blood was collected from the right atrium between 8 am and 11 am and transferred to 1.5 mL low-binding tubes. Following blood collection, mice were subsequently perfused intracardiacally with phosphate-buffered saline (PBS), followed by 4% paraformaldehyde (PFA). The brains were extracted and post-fixed overnight in 4% PFA at 4°C. They were subsequently transferred to a 15% sucrose solution in PBS for 1 day, followed by a switch to a 30% sucrose solution for another day. Brains were then frozen using OCT compound and stored at −80°C until sectioning.

### 2.4 ThioS staining

Coronal sections were obtained with a sliding microtome at 30 μm. Sections containing the prelimbic cortex (PrL) (approximately 1.6–2.5 mm anterior from bregma), anterior insula (AI) (approximately 1.2–2.0 mm anterior from bregma), and dentate gyrus of the hippocampus (DG) (approximately 1.3–2.3 mm posterior from bregma) were mounted onto ColorFrost Plus slides (Fisher Scientific) and allowed to dry overnight. Slides were then submerged for 1 min each in 70 and 80% ethanol prior to incubation for 15 min in 1% Thioflavin S (Acros Organics) in 80% ethanol. Slides were then washed 1 min each in 80 and 70% ethanol, then submerged in two changes of deionized water prior to coverslipping with ProLong Gold mounting medium (Thermo Fisher). Epifluorescent images (EVOS M5000 imaging system, Thermo Fisher) were taken from three sections per animal for each brain region at 20X (PrL and AI) or 10X (DG) magnification. Fluorescence data were thresholded using the “Triangle” autothreshold function of ImageJ (Zack et al., 1977) to account for varying background fluorescence. The number, total area, and average size of amyloid plaques were analyzed using the Analyze Particles function of ImageJ software. The average of counting was reported for each animal.

### 2.5 Corticosterone assay

The collected blood was left at room temperature for 1 h, followed by centrifugation for 15 min at 4°C at 3,000 rpm to separate the supernatant, which was then transferred into 1.5 mL tubes and stored in a −80°C freezer. Serum corticosterone levels were measured using a commercial ELISA kit (Cayman #501320) as described previously with minor modifications (Niwa et al., 2013, 2024).

### 2.6 Statistical analysis

The normality and homogeneity of variances were assessed using the Shapiro–Wilk and Levene’s tests, respectively. In cases where the data met the assumptions of normality and homogeneity of variances, a two-way ANOVA was conducted, treating late adolescent stress and pregnancy/delivery as independent factors. Subsequently, a Bonferroni *post hoc* test was conducted for pairwise comparisons across all groups, with adjustments made for multiple comparisons. Conversely, for data that did not meet these assumptions, a Mann–Whitney *U* test was employed for each independent factor, without correction for multiple comparisons, as this test is suited for comparing two groups. Significance was determined at the *p* < 0.05 level. For correlations, we used linear regression and reported the *R*^2^ values. Additional statistical details can be found in Supplementary Table S1. Data is presented as mean ± standard error of the mean (SEM), and our statistical analyses were conducted using GraphPad Prism 8 and SPSS 23.

## 3 Results

### 3.1 Late adolescent social isolation accelerates AD-related novelty recognition deficits in 5xFAD dams

**Figure 2:**
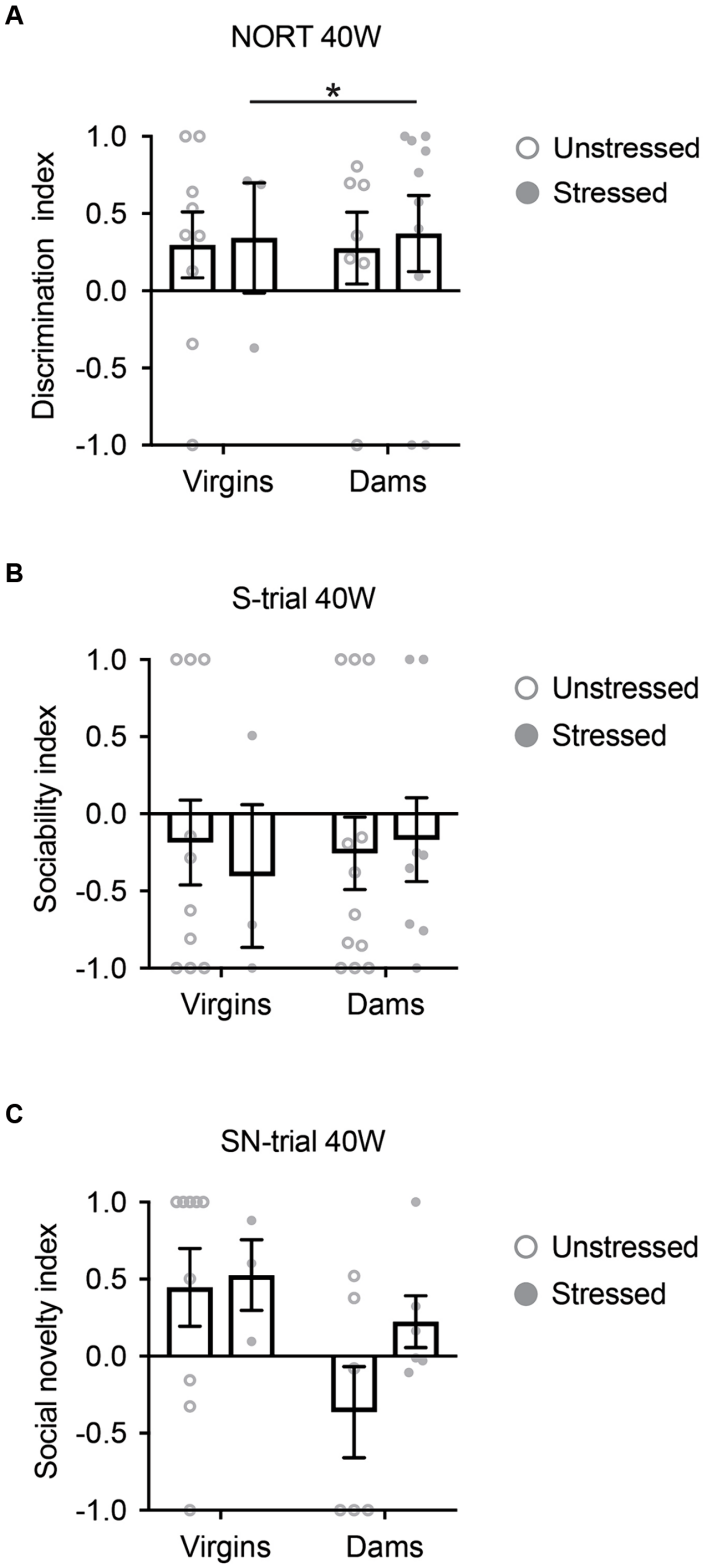
Cognitive behavioral changes in 5xFAD mice at 40 weeks of age. **(A)** Regardless of pregnancy and delivery history, both non-stressed and stressed 5xFAD mice exhibited behavioral deficits in the novelty object recognition test (NORT). **(B)** In the social interaction test (SIT), both non-stressed and stressed 5xFAD mice showed behavioral deficits in the sociability-trial (S-trial), irrespective of their pregnancy and delivery history. **(C)** In SIT, both non-stressed and stressed 5xFAD mice demonstrated behavioral deficits in the social novelty-trial (SN-trial), regardless of their pregnancy and delivery history. All data were non-normally distributed and analyzed using the Mann–Whitney *U* test. All comparisons were non-significant (*p* > 0.05), except stressed virgins vs. stressed dams for NORT (^*^p = 0.034). *N* = 3–12. Data are presented as mean ± SEM. See Supplementary Table S1 for details on the sample size and statistical analyses.

We first conducted NORT and SIT to examine the effects of late adolescent stress on non-social and social cognitive behavior, particularly focusing on novelty recognition, in 5xFAD mice. Results revealed no differences in either NORT or SIT at 40 weeks of age in both non-stressed and stressed 5xFAD mice, regardless of their pregnancy and delivery history (Figures 2A–C). Notably, a significant portion of the 5xFAD mice at 40 weeks of age had already perished by the time the behavioral experiments were conducted (Supplementary Figure S2). Furthermore, among those that survived until 40 weeks, a substantial portion displayed freezing behavior during the behavioral tests, leading to their exclusion from the analysis. This resulted in a limited sample size for some groups (Figure 2).

**Figure 3:**
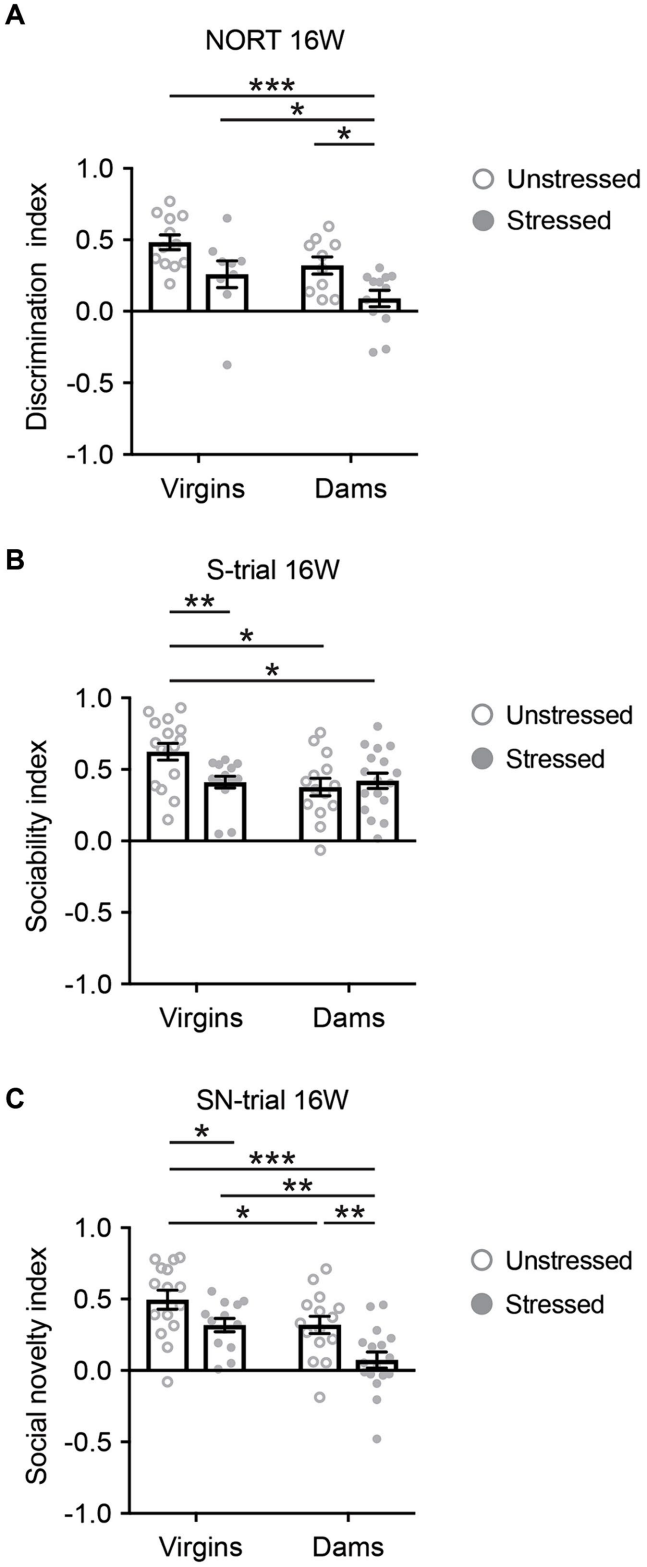
Cognitive behavioral changes in 5xFAD mice at 16 weeks of age. **(A)** Only stressed 5xFAD dams displayed behavioral deficits in novelty object recognition when compared to the other three groups. Mann–Whitney *U* test: Unstressed virgins vs. stressed dams, ^***^p < 0.001; stressed virgins vs. stressed dams, ^*^p < 0.05; unstressed dams vs. stressed dams, ^*^p < 0.05. **(B)** During the S-trial of SIT, stressed 5xFAD virgins, unstressed 5xFAD dams, and stressed 5xFAD dams showed deficits in sociability when compared to unstressed virgins. Mann–Whitney *U* test: Unstressed virgins vs. stressed virgins, ^**^p < 0.01; unstressed virgins vs. unstressed dams, ^*^p < 0.05; unstressed virgins vs. stressed dams, ^*^p < 0.05. **(C)** In the SN-trial of SIT, stressed 5xFAD dams exhibited an accelerated behavioral deficit in social novelty recognition compared to the other three groups. Two-way ANOVA: For interaction, *F*_(3,56)_ = 0.325, *p* = 0.571, partial *η*^2^ = 0.006; for stress, *F*_(1,56)_ = 12.972, *p* = 0.001, partial *η*^2^ = 0.188; for pregnancy/delivery, *F*_(1,56)_ = 12.842, *p* = 0.001, partial *η*^2^ = 0.187. Bonferroni: Unstressed virgins vs. stressed virgins, ^*^p < 0.05; stressed virgins vs. stressed dams, ^**^p < 0.01; unstressed dams vs. stressed dams, ^**^p < 0.01; unstressed virgins vs. stressed dams, ^***^p < 0.001. *N* = 9–17. Data are presented as mean ± SEM. See Supplementary Table S1 for details on the sample size and statistical analyses.

In contrast, most 5xFAD mice subjected to behavioral experiments at 16 weeks survived, and none exhibited freezing behavior during the tests. At 16 weeks, only stressed 5xFAD dams displayed behavioral deficits in NORT when compared to the other three groups (Figure 3A). During the NORT training phase at 16 weeks, differences were observed between unstressed virgins and dams, regardless of stress exposure, whereas no differences were observed among the four groups at 40 weeks (Supplementary Figure S3). During the S-trial of SIT at 16 weeks, stressed 5xFAD virgins, unstressed 5xFAD dams, and stressed 5xFAD dams showed deficits in sociability when compared to unstressed virgins (Figure 3B). Notably, in the SN-trial of SIT at 16 weeks, stressed 5xFAD dams exhibited an accelerated behavioral deficit in social novelty recognition compared to the other three groups (Figure 3C).

These findings suggest that late adolescent social isolation, in conjunction with pregnancy and delivery, accelerated the onset of AD-related behavioral deficits in non-social and social novelty recognition, but not in sociability.

### 3.2 Impaired novelty recognition in 5xFAD dams is correlated with increased corticosterone levels, but not with *β*-amyloid plaques

To understand how adolescent stress affects cognitive behaviors in 5xFAD dams, we next examined the effects of adolescent stress on hypothalamic–pituitary–adrenal (HPA) axis regulation and AD-related neuropathological changes in 5xFAD mice at 16 and 40 weeks of age. We evaluated serum corticosterone levels and the presence of *β*-amyloid plaques in key brain regions responsible for novelty recognition, including AI, PrL, and DG.

**Figure 4:**
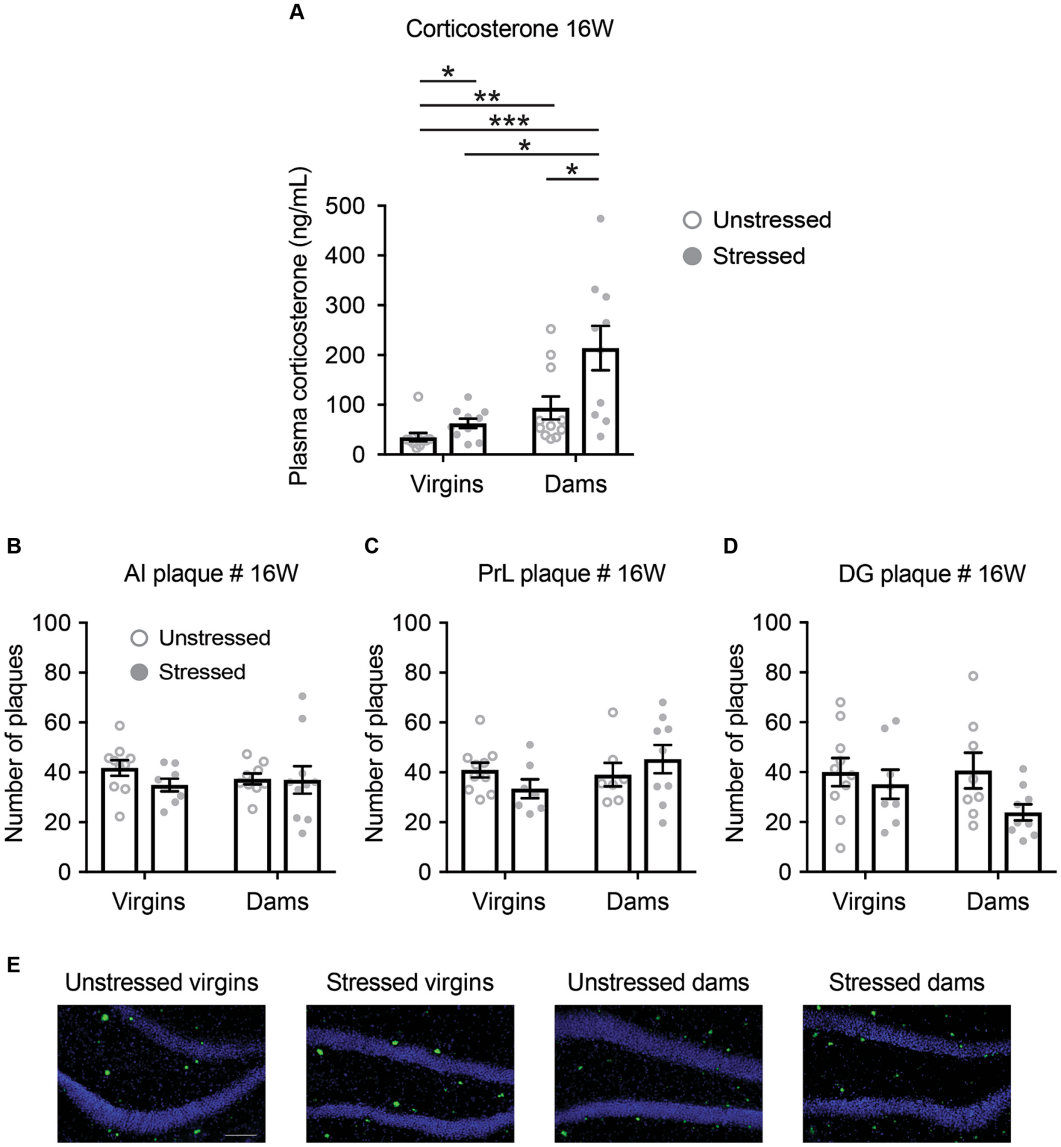
Changes in serum corticosterone levels and the quantity of *β*-amyloid plaques in 5xFAD mice at 16 weeks of age. **(A)** Serum corticosterone levels increased in response to both late adolescent social isolation and pregnancy/delivery, with a notably accelerated rise when both conditions co-occurred. Mann–Whitney *U* test: Unstressed virgins vs. stressed virgins, ^*^p < 0.05; unstressed virgins vs. unstressed dams, ^**^p < 0.01; unstressed virgins vs. stressed dams, ^***^p < 0.001; stressed virgins vs. stressed dams, ^**^p < 0.05; unstressed dams vs. stressed dams, ^*^p < 0.05. **(B–D)** Late adolescent social isolation and/or pregnancy/delivery did not affect the number of *β*-amyloid plaques in the anterior insula (AI; **B**), prelimbic cortex (PrL; **C**) and dentate gyrus of the hippocampus (DG; **D**), as evaluated by a Two-Way ANOVA, except for the comparison between unstressed virgins vs. stressed dams using Bonferroni adjustments (*p* = 0.027). **(E)** Representative images of *β*-amyloid plaques in the dentate gyrus of the hippocampus. *p* > 0.05 for all main factors and interaction. *N* = 7–11. Data are presented as mean ± SEM. See Supplementary Table S1 for details on the sample size and statistical analyses.

Following the final behavioral testing at 16 weeks of age, serum corticosterone levels were increased in response to both late adolescent social isolation and pregnancy/delivery (Figure 4A). Notably, the combination of late adolescent social isolation and pregnancy/delivery resulted in a further increase in serum corticosterone levels (Figure 4A). Interestingly, serum corticosterone levels across all four groups at 40 weeks of age were similar to those observed in stressed dams at 16 weeks of age (Supplementary Figure S4A), suggesting that late adolescent stress accelerated the increase in corticosterone levels at 16 weeks of age.

There were no statistically significant differences among the four groups in the number, percentage area, and size of *β*-amyloid plaques in AI, PrL, and DG at both 16 and 40 weeks of age, except for the comparison between unstressed virgins and stressed virgins regarding AI area and DG area at 40 weeks of age, the comparison between unstressed virgins and unstressed dams regarding AI size at 40 weeks of age, and the comparison between stressed virgins and stressed dams regarding DG area and size at 16 weeks of age (Figures 4B–E and Supplementary Figures S4B–D, S5).

**Figure 5:**
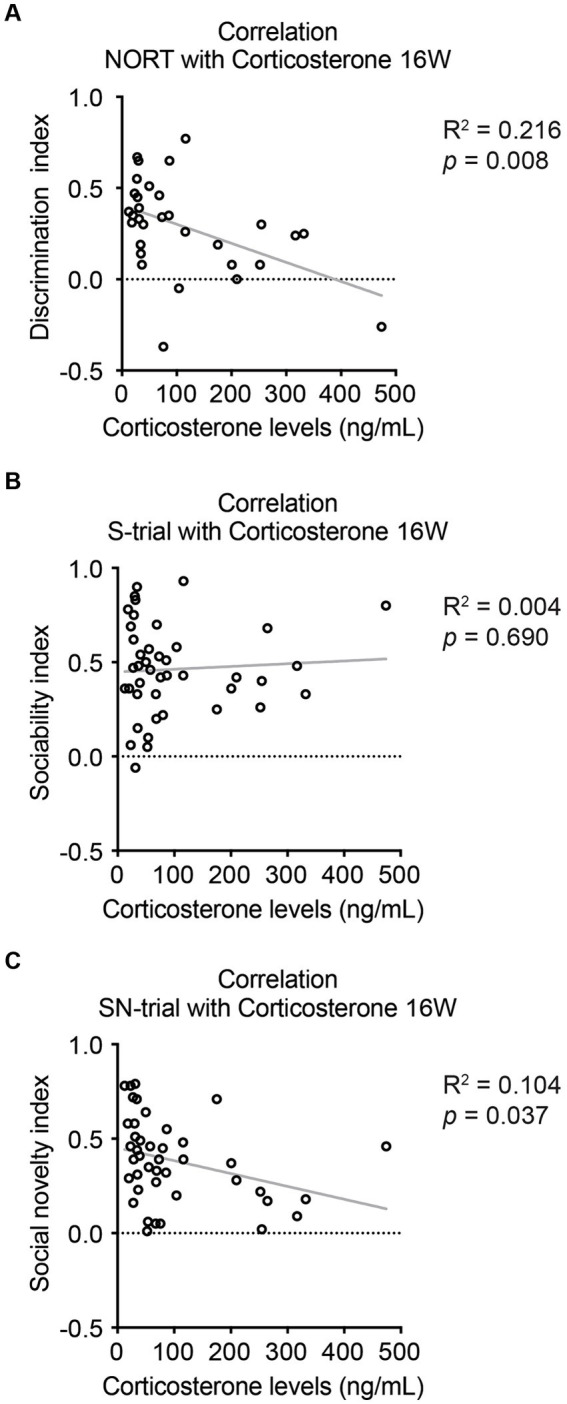
Correlations between corticosterone levels and cognitive behaviors in 5xFAD mice at 16 weeks of age. **(A)** Serum corticosterone levels were found to be negatively correlated with the novelty recognition indexes for NORT. **(B)** There was no significant correlation between corticosterone levels and sociability indexes for SIT during the S-trial. **(C)** A negative correlation was observed between corticosterone levels and sociability indexes for SIT during the SN-trial. *N* = 7–11. *R*^2^ and *p-*values were obtained from Pearson’s correlation test for all graphs. See Supplementary Table S1 for details on the sample size and statistical analyses.

Subsequently, we explored the correlations between behavioral alterations and molecular changes. We observed negative correlations between corticosterone levels and the behavioral indexes for NORT and the SN-trial of SIT, but not for the S-trial of SIT (Figure 5). Conversely, we found no correlations between the number of *β*-amyloid plaques and the behavioral scores for NORT and SIT S- and SN-trials (Supplementary Figure S6). These findings suggest a potential link between HPA axis dysregulation and novelty recognition impairments in stressed 5xFAD dams, while indicating no direct link with *β*-amyloid plaques.

## 4 Discussion

The present study investigated the effects of late adolescent social isolation combined with pregnancy and delivery on novelty recognition deficits in 5xFAD female mice. Drawing upon prior research establishing a link between corticosterone levels and behavioral outcomes (Kim et al., 2013; Henckens et al., 2016), correlation analyses were conducted. Our findings indicate that late adolescent social isolation, when coupled with pregnancy and delivery, can accelerate the onset of novelty recognition deficits, accompanied by increased corticosterone levels. Surprisingly, we did not see correlations between *β*-amyloid plaques and behavioral deficits, emphasizing the intricate relationship between stress and cognitive impairments associated with AD. This suggests that dysfunction in the HPA axis might have a more direct association with behavioral impairment than *β*-amyloid plaques.

Ongoing debate surrounds the involvement of *β*-amyloid plaques in AD pathophysiology (Piller, 2022; Zhang et al., 2023). While the present study may present evidence countering the “amyloid cascade hypothesis” of AD, we acknowledge the inherent limitations in our methods and data, preventing us from definitively refuting the role of *β*-amyloid plaques in AD. For instance, prior research has demonstrated that soluble forms of *β*-amyloid protein correlate more strongly with AD-related cognitive deficits than plaques (Milligan Armstrong et al., 2021). Therefore, our mouse model may feature elevated levels of soluble *β*-amyloid, prompting further exploration in future studies. Nevertheless, our findings suggest that other mechanisms, such as HPA axis dysregulation, may play a more prominent role in stress-induced cognitive impairment.

Pregnancy and the postpartum period are recognized as sensitive periods for women, both hormonally and psychologically. Our study delves into the intersection of stress before pregnancy-particularly in the context of social isolation during late adolescence-, pregnancy/delivery, and subsequent risk of AD later in life. This is particularly pertinent considering the disproportionate risk of AD faced by women compared to men, with pregnancy and hormonal fluctuations during reproductive years potentially serving as significant contributing factors.

Our study highlights the potential impact of stress during critical developmental periods, such as adolescence, on cognitive function and AD risk in females. Stress, particularly early life stress, is recognized as a risk factor for AD in humans as well (Heim and Nemeroff, 2001; Donley et al., 2018). AD patients often exhibit elevated levels of corticosteroids in plasma and cerebrospinal fluid (Milligan Armstrong et al., 2021). Epigenetic modification of HSD11B1, an enzyme involved in cortisol metabolism, has been associated with late-onset AD and is affected by early life stress (Lemche, 2018). These findings underscore the importance of investigating the relationship between stress, glucocorticoid levels, and AD pathophysiology in the context of pregnancy and the postpartum period, particularly in women.

The behavioral changes we observed cannot solely be attributed to short-term factors. Notably, social isolation was applied during late adolescence, spanning from 5 to 8 weeks of age. Subsequently, there was a minimum additional interval of 3 weeks between mating and delivery. Dams were then tested approximately 5 weeks after delivery. Thus, the behavioral effects were evaluated at least 5 weeks after the last significant stressor, which was delivery, and 8 weeks after the last isolation exposure. We posit that these observed effects primarily indicate long-term consequences. However, it is essential to acknowledge that a different experimental design would be necessary to thoroughly assess this assertion.

In the present study, we did not observe significant effects of parturition on behavior associated with AD. The relationship between parity and AD risk remains complex and not fully understood (Galea et al., 2018; Deems and Leuner, 2020; Jung et al., 2020). While some research suggests that multiple childbirths may offer a protective effect, our focus lies on the impact of one-time delivery, which has shown no effect in both our present and published studies (Kin et al., 2023; Niwa et al., 2024,). This potential protective effect is believed to be linked to hormonal changes, particularly increased estrogen during pregnancy. Nonetheless, findings in the literature are inconclusive, with some studies indicating no significant association. Further investigation is essential to gain a deeper understanding of the underlying mechanisms involved, considering genetic and lifestyle factors, especially in the context of female-specific AD risk factors.

This study has several important limitations. Firstly, our 40-week cohort had a small sample size, which could impact our statistical analyses. Additionally, while mice that died before the 40-week behavioral experiments or exhibited freezing behavior during the experiments were not included in the analysis, it cannot be ruled out that these mice may have shown even more severe behavioral deficits. Secondly, we collected blood samples and conducted brain perfusions 1 day after the stressful behavioral experiments rather than immediately afterward. While we interpret that prolonged, rather than acute, elevation in corticosterone levels may underlie impaired novelty recognition behaviors in stressed dams, we also acknowledge the need for a cautious interpretation due to the limited nature of our findings and methodological considerations. More frequent sampling and detailed examination of glucocorticoid signaling, along with other reproductive steroid hormones and diverse brain regions/circuits, could yield significant benefits. Thirdly, we focused our study solely on 5xFAD mice, making it challenging to conclude the effects of 5xFAD mutations. Exploring a comparison with a wild-type strain to assess the effects of 5xFAD mutations would be an intriguing avenue for future research. Fourthly, we only assessed subjects with novelty discrimination tasks in the present study. To connect our current findings with the broader relevance of stress-induced cognitive impairments, further behavioral assessments related to cognition are necessary in future studies. Despite these limitations, we believe our study provides valuable data indicating the important role of adolescent stress in accelerating AD pathology in the context of pregnancy and the postpartum period.

While our study provides novel insights into the intricate relationship among stress, hormonal factors, and cognitive deficits in the context of AD, particularly in females, and highlights the importance of considering these factors in AD research and therapeutic development, it is worth noting that this interplay may not be exclusive to AD mouse models. Further research, both in animal models and in human studies, will be necessary to fully elucidate the intricate relationship between stress and AD. Building on our findings, this continued investigation will contribute to a deeper understanding of the underlying mechanisms and provide insights into how stress may affect cognitive function in human AD, particularly in women. By elucidating the mechanisms by which stress influences cognitive function via HPA axis regulation, especially during vulnerable periods such as adolescence, pregnancy, and childbirth, we can not only enhance our understanding of AD pathogenesis but also inform preventive strategies and interventions aimed at mitigating cognitive decline across the lifespan. Therefore, it is imperative to investigate how stress interacts with genetic predispositions and life experiences to modulate cognitive health, with the ultimate goal of developing targeted approaches for the prevention and treatment of cognitive impairment, including AD.

In conclusion, this study contributes to our comprehension of the complex interplay between stress, cognitive deficits, and AD in the context of women’s health. It highlights the importance of considering not only the traditional markers of AD, such as *β*-amyloid plaques, but also the hormonal and stress-related aspects of the disease, particularly in females. These findings may contribute to the broader discourse concerning AD risk factors and potential avenues for intervention and prevention.

## Data availability statement

The raw data supporting the conclusions of this article will be made available by the authors, without undue reservation.

## Ethics statement

The animal study was approved by UAB Institutional Animal Care and Use Committee. The study was conducted in accordance with the local legislation and institutional requirements.

## Author contributions

OL: Investigation, Writing – original draft, Writing – review & editing. JF-O: Formal analysis, Writing – original draft, Writing – review & editing. SK: Formal analysis, Writing – original draft, Writing – review & editing. LA: Investigation, Writing – original draft, Writing – review & editing. SR: Writing – original draft, Writing – review & editing. S-iK: Supervision, Writing – original draft, Writing – review & editing. AA: Formal analysis, Investigation, Writing – original draft, Writing – review & editing. MN: Conceptualization, Data curation, Formal analysis, Funding acquisition, Investigation, Methodology, Project administration, Resources, Software, Supervision, Validation, Visualization, Writing – original draft, Writing – review & editing.
