# Supplementary material for: Adolescent stress accelerates postpartum novelty recognition impairment in 5xFAD mice
Source: Front Neurosci. 2024 May 15;18:1366199. doi: 10.3389/fnins.2024.1366199 (PMC11133596; doi:10.3389/fnins.2024.1366199)
Supplement: Supplementary file 1 [file Data_Sheet_1.PDF]

Supplemental material for

Adolescent Stress Accelerates Postpartum Novelty Recognition Impairment In 5xFAD Mice

Owen Leitzel[1,2,+], Jose Francis-Oliveira[1,+], Shaimaa M. Khedr[1], Lila Ariste[1,3], Stefanie Robel[2], Shin-ichi Kano[1,4], Andrew Arrant[5], Minae Niwa[1,4,6*]

[1] Department of Psychiatry and Behavioral Neurobiology, University of Alabama at Birmingham, Birmingham, AL, USA

[2] Department of Cell, Developmental and Integrative Biology, University of Alabama at Birmingham, Birmingham, AL, USA

[3] Department of Biology, Adelphi University, Garden City, NY, USA

[4] Department of Neurobiology, University of Alabama at Birmingham, Birmingham, AL, USA

[5] Department of Neurology, University of Alabama at Birmingham, Birmingham, AL, USA

[6] Department of Biomedical Engineering, University of Alabama at Birmingham, Birmingham, AL, USA

[+]These authors contributed equally to this work and share first authorship.

***Correspondence:** mniwa@uabmc.edu

This PDF file includes: Supplemental Figures 1 to 6

Supplemental Table 1

Fig. S1

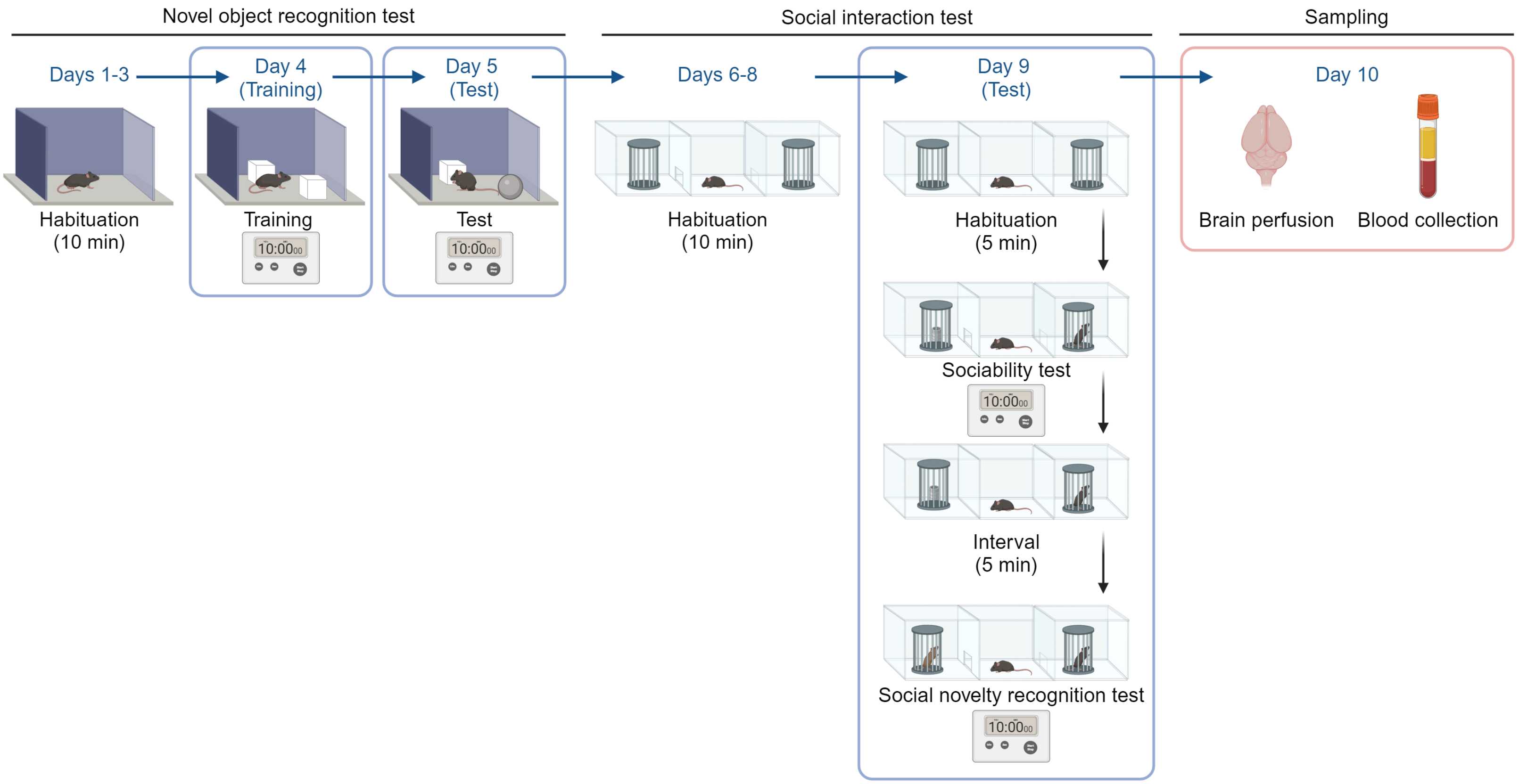
Novel object recognition test
Social interaction test
Sampling
Days 1-3
Day 4
(Training)
Day 5
(Test)
Days 6-8
Day 9
(Test)
Day 10
Habituation
(10 min)
Training
10:00 00
Test
10:00 00
Habituation
(10 min)
Habituation
(5 min)
Sociability test
10:00 00
Interval
(5 min)
Social novelty recognition test
10:00 00
Brain perfusion
Blood collection

Figure S1. Experimental Design. Two distinct cohorts were prepared for experiments, with one at 16 weeks and the other at 40 weeks of age. Each animal underwent a multi-day behavioral testing regimen over a total of 9 days. On the 10th day, brain perfusion and blood collection procedures were conducted. This figure was created with BioRender.com.

Fig. S2

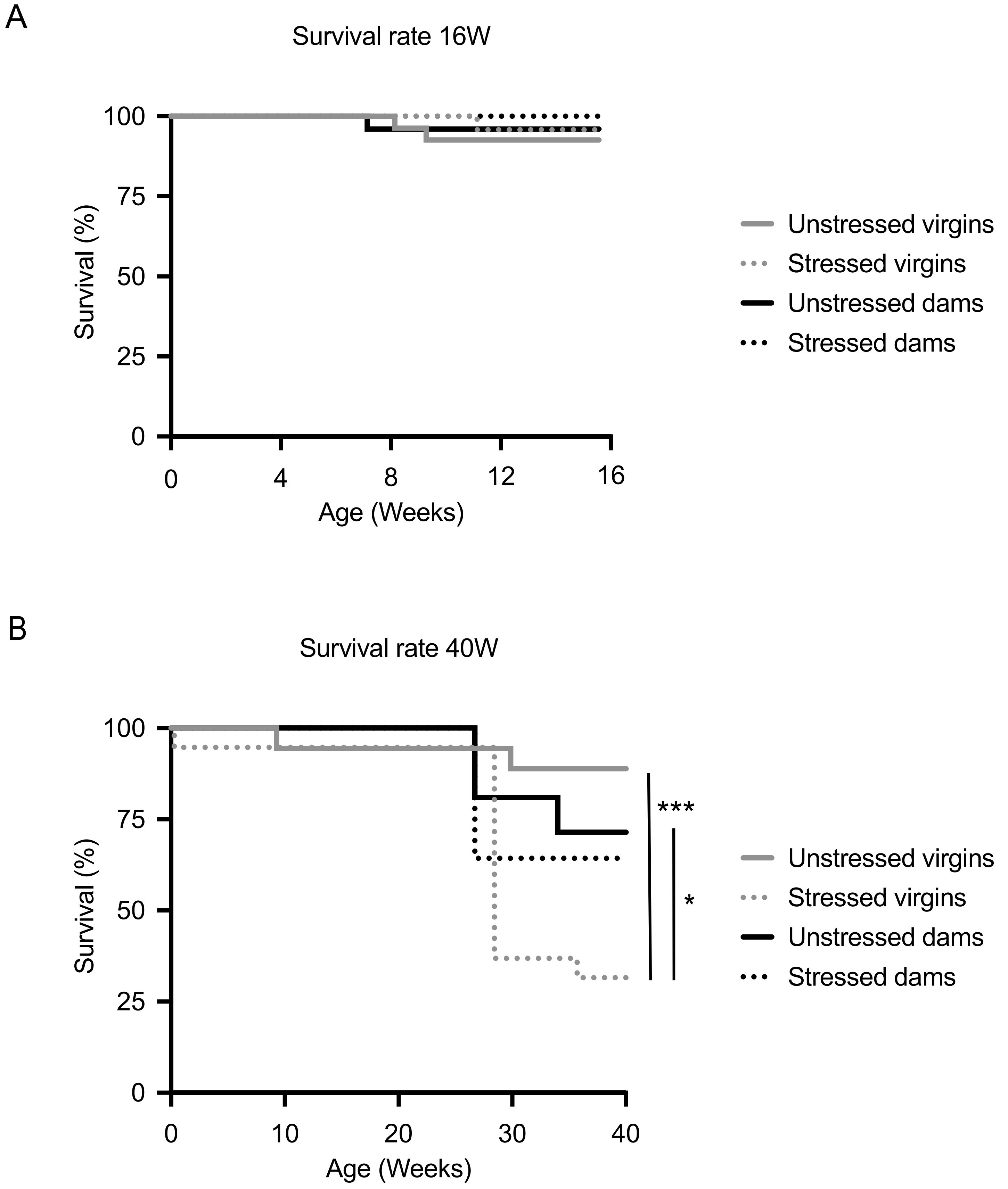
A
Survival rate 16W
100
75
50
25
0
Survival (%)
0
4
8
12
16
Age (Weeks)
Unstressed virgins
Stressed virgins
Unstressed dams
Stressed dams
B
Survival rate 40W
100
75
50
25
0
Survival (%)
0
10
20
30
40
Age (Weeks)
***
*
Unstressed virgins
Stressed virgins
Unstressed dams
Stressed dams

Figure S2. Adolescent stress-induced decrease in survival of 5xFAD mice. A. Survival curve for the 5xFAD 16-week cohort. **B.** Survival curve for the 5xFAD 40-week cohort. Survival rate for stressed virgins was significantly lower than for the unstressed groups. Differences between groups were detected through the Kaplan-Meier analysis. See **Table S1** for details on the sample size and statistical analyses.

Fig. S3

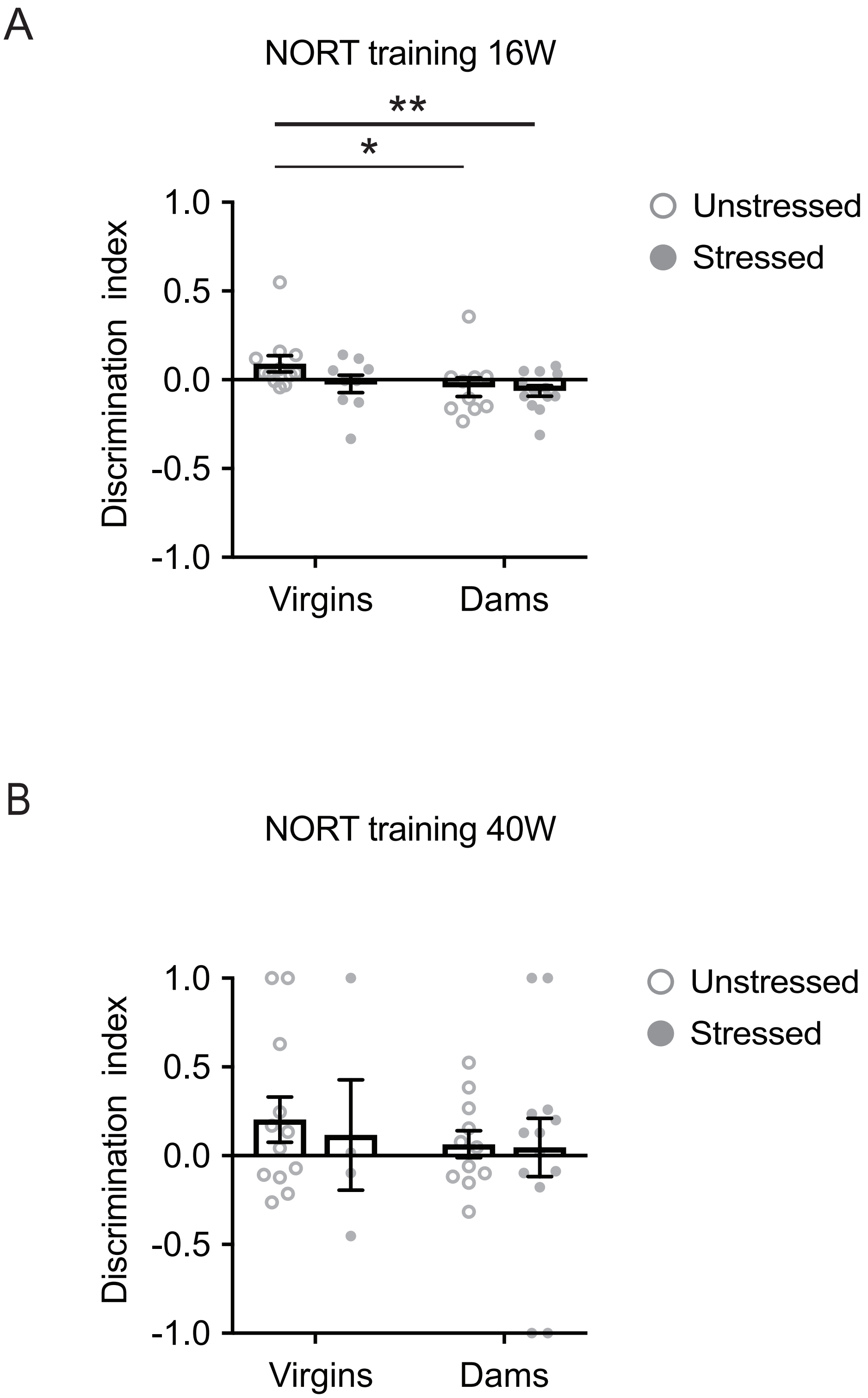
A
NORT training 16W
**
*
Discrimination index
1.0
0.5
0.0
-0.5
-1.0
Virgins
Dams
Unstressed
Stressed
B
NORT training 40W
Discrimination index
1.0
0.5
0.0
-0.5
-1.0
Virgins
Dams
Unstressed
Stressed

Figure S3. Cognitive behavioral training session with two identical objects in NORT in 5xFAD mice at 16 and 40 weeks of age. A-B. At 16 weeks, differences were observed between unstressed virgins and dams, both with and without stress. No significant differences were observed among the four groups during the NORT training phase at 40 weeks. All data, which were non-normally distributed, were analyzed using the Mann-Whitney U test, comparing all groups. Unstressed virgins vs stressed dams, ***$p < 0.001$. N=4-13. Data are presented as mean ± SEM. See **Table S1** for details on the sample size and statistical analyses.

Fig. S4

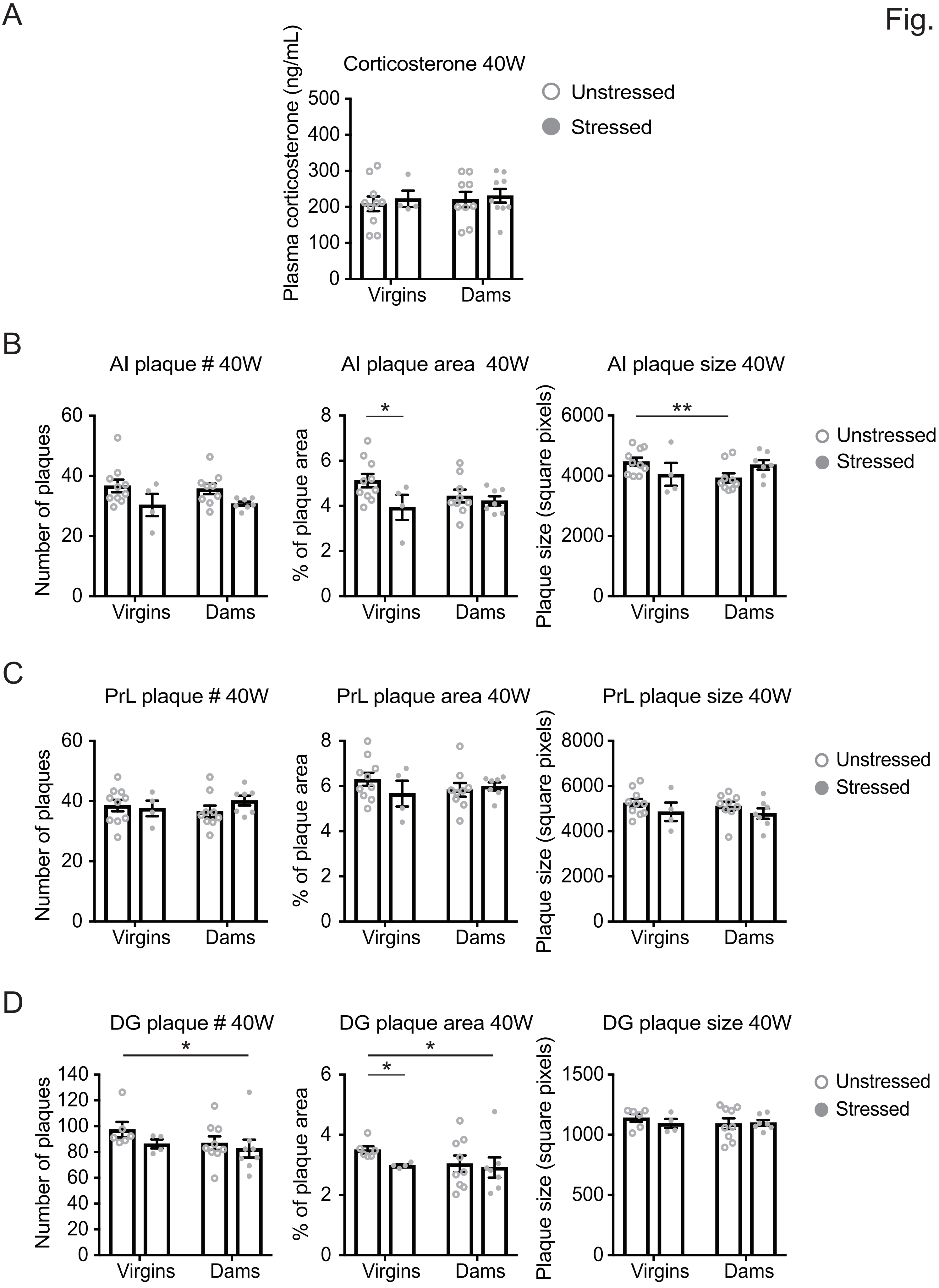

A
Corticosterone 40W
Plasma corticosterone (ng/mL)
500
400
300
200
100
0
Virgins
Dams
Unstressed
Stressed
B
AI plaque # 40W
Number of plaques
60
40
20
0
Virgins
Dams
AI plaque area 40W
% of plaque area
8
6
4
2
0
*
AI plaque size 40W
Plaque size (square pixels)
6000
4000
2000
0
**
Unstressed
Stressed
C
PrL plaque # 40W
PrL plaque area 40W
PrL plaque size 40W
8000
D
DG plaque # 40W
140
120
100
80
60
40
20
DG plaque area 40W
DG plaque size 40W
1500
1000
500

Figure S4. No changes in serum corticosterone levels and β-amyloid plaques in 5xFAD mice at 40 weeks of age. A. At 40 weeks of age, serum corticosterone levels were increased (200 ng/mL) in all four groups, with no significant differences between them (Mann Whitney). **B.** Adolescent social isolation and pregnancy/delivery may have tendency to reduce β-amyloid plaques in the AI at 40 weeks of age, but the differences were not consistent among groups and parameters (Two-Way ANOVA for count and area, and Mann Whitney for size). **C.** β-amyloid plaques in the PrL at 40 weeks of age were not different between four groups (Two-Way ANOVA). **D.** In the DG at 40 weeks of age, stressed dams had a reduction in counting and area of β-amyloid plaques (Mann Whitney). N=4-10. Data are presented as mean ± SEM. See **Table S1** for details on the sample size and statistical analyses.

Fig. S5

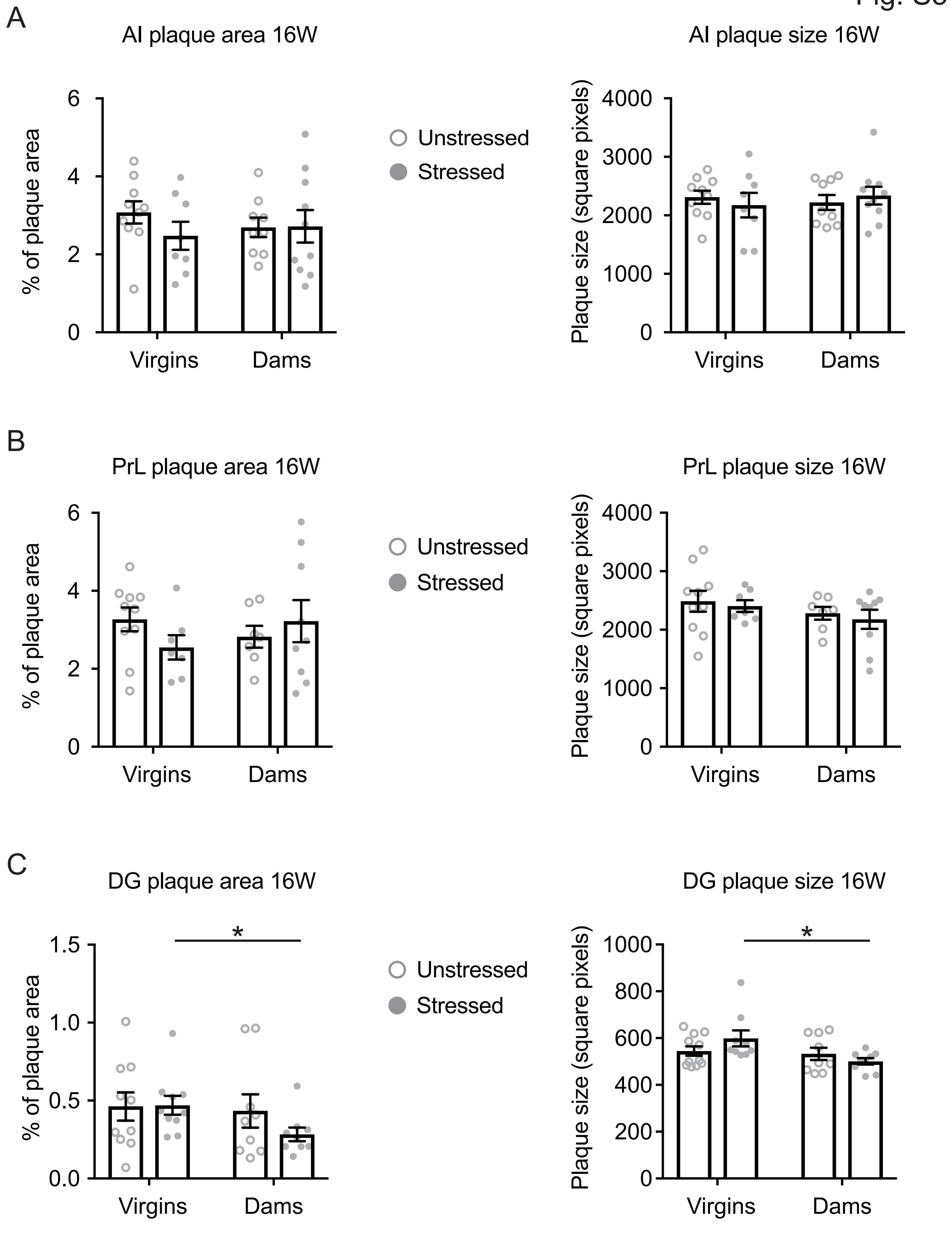
A
AI plaque area 16W
% of plaque area
0
2
4
6
Virgins
Dams
Unstressed
Stressed
AI plaque size 16W
Plaque size (square pixels)
0
1000
2000
3000
4000
Virgins
Dams
B
PrL plaque area 16W
% of plaque area
0
2
4
6
Virgins
Dams
Unstressed
Stressed
PrL plaque size 16W
Plaque size (square pixels)
0
1000
2000
3000
4000
Virgins
Dams
C
DG plaque area 16W
% of plaque area
0.0
0.5
1.0
1.5
*
Virgins
Dams
Unstressed
Stressed
DG plaque size 16W
Plaque size (square pixels)
0
200
400
600
800
1000
*
Virgins
Dams

Figure S5. Unchanged quantity and size of β-amyloid plaques in 5xFAD mice at 16 weeks of age. A-C. Adolescent social isolation and/or pregnancy/delivery had no impact on the percentage of area and size of β-amyloid plaques in AI (**A**), PrL (**B**), and DG (**C**). Two-way ANOVA and Mann Whitney tests were conducted, and all p values were > 0.05, except the comparison between stressed virgins vs stressed dams for DG area and size (Mann Whitney, $p = 0.027$ for area and $p = 0.021$ for size). N=7-10. Data are presented as mean ± SEM. See **Table S1** for details on the sample size and statistical analyses.

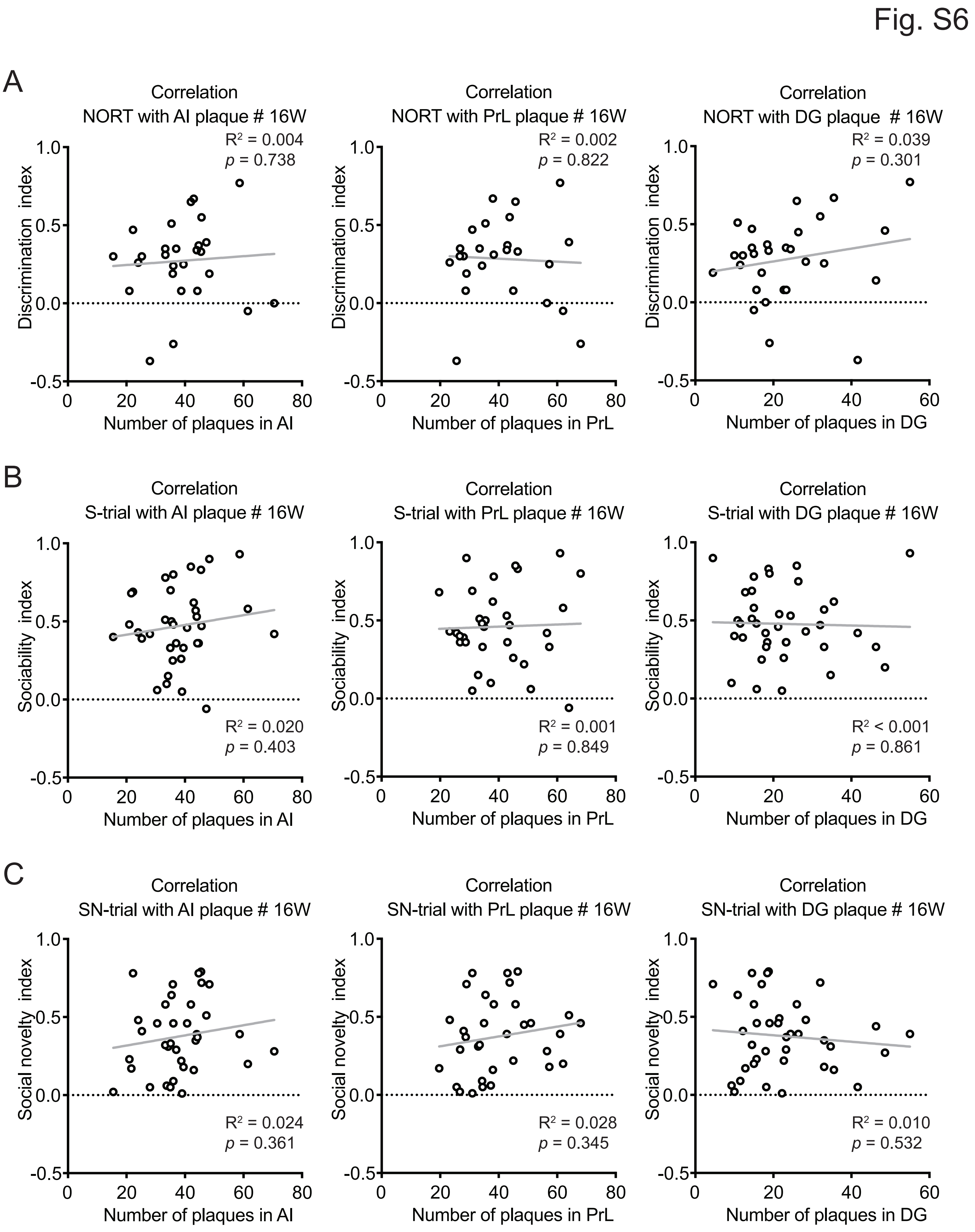
Fig. S6
A
Correlation
NORT with AI plaque # 16W
R^2 = 0.004
p = 0.738
Discrimination index
Number of plaques in AI
Correlation
NORT with PrL plaque # 16W
R^2 = 0.002
p = 0.822
Discrimination index
Number of plaques in PrL
Correlation
NORT with DG plaque # 16W
R^2 = 0.039
p = 0.301
Discrimination index
Number of plaques in DG
B
Correlation
S-trial with AI plaque # 16W
R^2 = 0.020
p = 0.403
Sociability index
Number of plaques in AI
Correlation
S-trial with PrL plaque # 16W
R^2 = 0.001
p = 0.849
Sociability index
Number of plaques in PrL
Correlation
S-trial with DG plaque # 16W
R^2 < 0.001
p = 0.861
Sociability index
Number of plaques in DG
C
Correlation
SN-trial with AI plaque # 16W
R^2 = 0.024
p = 0.361
Social novelty index
Number of plaques in AI
Correlation
SN-trial with PrL plaque # 16W
R^2 = 0.028
p = 0.345
Social novelty index
Number of plaques in PrL
Correlation
SN-trial with DG plaque # 16W
R^2 = 0.010
p = 0.532
Social novelty index
Number of plaques in DG

Figure S6. No correlations between β-amyloid plaques and cognitive behaviors in 5xFAD mice at 16 weeks of age. A. No correlations were evident between β-amyloid plaques in the AI, PrL and DG regions and the novelty recognition indexes for NORT. **B.** No correlations were found between β-amyloid plaques in the AI, PrL and DG and the sociability indexes for the S-trial of SIT. **C.** No correlations were observed between β-amyloid plaques in the AI, PrL and DG and the novelty recognition indexes for the SN-trial of SIT. N=7-10. R^2 and *p* values were obtained from Pearson's correlation test for all graphs.

Supplementary Table 1. Statistical Analysis

Statistical tests performed for each data set are indicated.
p values considered significant and used to interpret the data are highlighted in yellow.
All p values are abbreviated to three digits.
UV = Unstressed Virgins
SV = Stressed Virgins
UD = Unstressed Dams
SD = Stressed Dams

Figure	Shapiro-Wilk test	Mann Whitney U test		
**Figure**	**Shapiro-Wilk test**	**Mann Whitney U test**		
**2A**	UV p = 0.324	UV vs UD	p = 1.000	U = 31.50
NORT	SV p = 0.034		r = 0.000	z = 0.00
40W	UD p = 0.041	SV vs SD	p = 0.034	U = 18.00
	SD p = 0.007		r = 0.141	z = 0.51
	**Sample Size**	UV vs SV	p = 0.864	U = 15.00
	UV n = 9		r = 0.080	z = 0.28
	SV n = 3	UD vs SD	p = 0.417	U = 44.00
	UD n = 7		r = 0.214	z = 0.88
	SD n = 10	UV vs SD	p = 0.661	U = 51.00
			r = 0.112	z = 0.49
**Figure**	**Shapiro-Wilk test**	**Mann Whitney U test**		
**2B**	UV p = 0.337	UV vs UD	p = 0.497	U = 15.50
S-trial	SV p = 0.014		r = 0.216	z = 0.72
40W	UD p = 0.064	SV vs SD	p = 0.872	U = 57.00
	SD p = 0.009		r = 0.043	z = 0.20
	**Sample Size**	UV vs SV	p = 0.692	U = 17.50
	UV n = 3		r = 0.120	z = 0.43
	SV n = 10	UD vs SD	p = 0.678	U = 42.50
	UD n = 8		r = 0.096	z = 0.43
	SD n = 12	UV vs SD	p = 0.762	U = 43.50
			r = 0.080	z = 0.31
**Figure**	**Shapiro-Wilk test**	**Mann Whitney U test**		
**2C**	UV p = 0.681	UV vs UD	p = 0.381	U = 5.00
SN-trial	SV p = 0.010		r = 0.344	z = 1.03
40W	UD p = 0.069	SV vs SD	p = 0.066	U = 11.50
	SD p = 0.054		r = 0.485	z = 1.88
	**Sample Size**	UV vs SV	p = 0.727	U = 16.00
	UV n = 3		r = 0.138	z = 0.48
	SV n = 9	UD vs SD	p = 0.310	U = 11.00
	UD n = 6		r = 0.326	z = 1.13
	SD n = 6	UV vs SD	p = 0.529	U = 21.50
			r = 0.220	z = 0.66
**Figure**	**Shapiro-Wilk test**	**Mann Whitney U test**		
**3A**	UV p = 0.639	UV vs UD	p = 0.080	U = 33.00
NORT	SV p = 0.107		r = 0.379	z = 1.78
16W	UD p = 0.316	SV vs SD	p = 0.034	U = 24.00
	SD p = 0.034		r = 0.455	z = 2.13
	**Sample Size**	UV vs SV	p = 0.058	U = 27.00
	UV n = 12		r = 0.419	z = 1.92
	SV n = 9	UD vs SD	p = 0.043	U = 29.00
	UD n = 10		r = 0.426	z = 2.04
	SD n = 13	UV vs SD	p < 0.001	U = 6.00

			r = 0.383	z = 0.38
**Figure**	**Shapiro-Wilk test**	**Mann Whitney U test**		
**3B**	UV p = 0.286	UV vs UD	p = 0.010	U = 60.00
S-trial	SV p = 0.010		r = 0.468	z = 2.56
16W	UD p = 0.946	SV vs SD	p = 0.512	U = 129.00
	SD p = 0.973		r = 0.121	z = 0.68
	**Sample Size**	UV vs SV	p = 0.004	U = 48.00
	UV n = 16		r = 0.511	z = 2.85
	SV n = 15	UD vs SD	p = 0.800	U = 127.00
	UD n = 14		r = 0.050	z = 0.28
	SD n = 17	UV vs SD	p = 0.016	U = 70.00
			r = 0.201	z = 0.21
**Figure**	**Shapiro-Wilk test**	**Two-Way ANOVA**		
**3C**	UV p = 0.250			
SN-trial	SV p = 0.503	Interaction $F(3,56)$ = 0.325	, p = 0.571 ,	partial η^2 = 0.006
16W	UD p = 0.977	Stress $F(1,56)$ = 12.972	, p = 0.001 ,	partial η^2 = 0.188
	SD p = 0.588	Pregnancy $F(1,56)$ = 12.842	, p = 0.001 ,	partial η^2 = 0.187
	**Levene test**			
	p = 0.543	post hoc (Bonferroni)		
	**Sample Size**	UV vs UD	p = 0.037	
	UV n = 15	SV vs SD	p = 0.005	
	SV n = 13	UV vs SV	p = 0.043	
	UD n = 15	UD vs SD	p = 0.003	
	SD n = 17	UV vs SD	p < 0.001	
**Figure**	**Shapiro-Wilk test**	**Mann Whitney U test**		
**4A**	UV p = <0.001	UV vs UD	p = 0.001	U = 110.00
Cort	SV p = 0.818		r = 0.693	z = 3.25
16W	UD p = 0.003	SV vs SD	p = 0.011	U = 83.00
	SD p = 0.537		r = 0.558	z = 2.50
	**Sample Size**	UV vs SV	p = 0.043	U = 84.00
	UV n = 11		r = 0.446	z = 2.04
	SV n = 10	UD vs SD	p = 0.017	U = 89.00
	UD n = 11		r = 0.522	z = 2.39
	SD n = 10	UV vs SD	p < 0.001	U = 106.00
			r = 0.784	z = 3.59
**Figure**	**Shapiro-Wilk test**	**Two-Way ANOVA**		
**4B**	UV p = 0.604			
AI Count	SV p = 0.737	Interaction $F(3,33)$ = 0.729	, p = 0.399 ,	partial η^2 = 0.022
16W	UD p = 0.807	Stress $F(1,33)$ = 0.929	, p = 0.342 ,	partial η^2 = 0.027
	SD p = 0.178	Pregnancy $F(1,33)$ = 0.098	, p = 0.757 ,	partial η^2 = 0.003
	**Levene test**			
	p = 0.165	post hoc (Bonferroni)		
	**Sample Size**	UV vs UD	p = 0.408	
	UV n = 10	SV vs SD	p = 0.709	
	SV n = 8	UV vs SV	p = 0.215	
	UD n = 9	UD vs SD	p = 0.937	
	SD n = 10	UV vs SD	p = 0.490	
**Figure**	**Shapiro-Wilk test**	**Two-Way ANOVA**		

**4C**<br>PrL Count<br>16W	UV p = 0.452<br>SV p = 0.328<br>UD p = 0.089<br>SD p = 0.539<br>**Levene test**<br>p = 0.077<br>**Sample Size**<br>UV n = 10<br>SV n = 7<br>UD n = 7<br>SD n = 9	Interaction $F(3,29) = 2.377$, $p = 0.134$, partial $\eta^2 = 0.076$<br>Stress $F(1,29) = 0.021$, $p = 0.886$, partial $\eta^2 = 0.001$<br>Pregnancy $F(1,29) = 1.269$, $p = 0.134$, partial $\eta^2 = 0.076$<br><br>post hoc (Bonferroni)<br>UV vs UD p = 0.769<br>SV vs SD p = 0.072<br>UV vs SV p = 0.238<br>UD vs SD p = 0.337<br>UV vs SD p = 0.104
**Figure**<br>**4D**<br>DG Count<br>16W	**Shapiro-Wilk test**<br>UV p = 0.224<br>SV p = 0.883<br>UD p = 0.434<br>SD p = 0.553<br>**Levene test**<br>p = 0.215<br>**Sample Size**<br>UV n = 11<br>SV n = 9<br>UD n = 9<br>SD n = 9	**Two-Way ANOVA**<br>Interaction $F(3,34) = 0.872$, $p = 0.357$, partial $\eta^2 = 0.025$<br>Stress $F(1,34) = 0.157$, $p = 0.694$, partial $\eta^2 = 0.005$<br>Pregnancy $F(1,34) = 1.968$, $p = 0.170$, partial $\eta^2 = 0.055$<br><br>post hoc (Bonferroni)<br>UV vs UD p = 0.736<br>SV vs SD p = 0.116<br>UV vs SV p = 0.699<br>UD vs SD p = 0.365<br>UV vs SD p = 0.027
**Figure**<br>**5A**<br>NORT<br>16W	**Linear Regression and Pearson's Correlation**<br>$F(1,29) = 7.969$, $p = 0.008$<br>$R^2 = 0.216$	
**Figure**<br>**5B**<br>S-trial<br>16W	**Linear Regression and Pearson's Correlation**<br>$F(1,40) = 0.161$, $p = 0.690$<br>$R^2 = 0.004$	
**Figure**<br>**5C**<br>SN-trial<br>16W	**Linear Regression and Pearson's Correlation**<br>$F(1,40) = 4.635$, $p = 0.037$<br>$R^2 = 0.104$	
**Figure**<br>**S2A**<br>Survival<br>16W	**Kaplan-Meier**<br>$\chi^2(3) = 1.854$, $p = 0.603$	
**Figure**<br>**S2B**<br>Survival<br>40W	**Kaplan-Meier**<br>$\chi^2(3) = 11.410$, $p = 0.009$<br><br>UV vs SV $p < 0.001$<br>UV vs SD p = 0.083<br>UV vs UD p = 0.210<br>UD vs SV p = 0.032<br>SD vs SV p = 0.162	

Figure	Test	Comparison		
**Figure**	**Shapiro-Wilk test**	**Mann Whitney U test**		
**S3A**	UV $p =$ 0.001	UV vs UD	$p =$ 0.011	$U =$ 22.00
NORT	SV $p =$ 0.289		$r =$ 0.534	$z =$ 2.51
Training	UD $p =$ 0.049	SV vs SD	$p =$ 0.301	$U =$ 43.00
16W	SD $p =$ 0.399		$r =$ 0.221	$z =$ 1.04
	**Sample Size**	UV vs SV	$p =$ 0.310	$U =$ 39.00
	UV $n =$ 12		$r =$ 0.233	$z =$ 1.07
	SV $n =$ 9	UD vs SD	$p =$ 0.738	$U =$ 71.00
	UD $n =$ 10		$r =$ 0.078	$z =$ 0.37
	SD $n =$ 13	UV vs SD	$p =$ 0.007	$U =$ 29.00
			$r =$ 0.532	$z =$ 2.66
**Figure**	**Shapiro-Wilk test**	**Mann Whitney U test**		
**S3B**	UV $p =$ 0.036	UV vs UD	$p =$ 0.740	$U =$ 60.00
NORT	SV $p =$ 0.395		$r =$ 0.077	$z =$ 0.37
Training	UD $p =$ 0.875	SV vs SD	$p =$ 0.871	$U =$ 28.00
40W	SD $p =$ 0.096		$r =$ 0.055	$z =$ 0.23
	**Sample Size**	UV vs SV	$p =$ 0.684	$U =$ 20.00
	UV $n =$ 12		$r =$ 0.122	$z =$ 0.49
	SV $n =$ 4	UD vs SD	$p =$ 0.910	$U =$ 74.00
	UD $n =$ 11		$r =$ 0.030	$z =$ 0.15
	SD $n =$ 13	UV vs SD	$p =$ 0.852	$U =$ 74.00
			$r =$ 0.042	$z =$ 0.21
**Figure**	**Shapiro-Wilk test**	**Mann Whitney U test**		
**S4A**	UV $p =$ 0.432	UV vs UD	$p =$ 0.905	$U =$ 47.00
Cort	SV $p =$ 0.282		$r =$ 0.044	$z =$ 0.16
40W	UD $p =$ 0.014	SV vs SD	$p =$ 0.825	$U =$ 20.00
	SD $p =$ 0.376		$r =$ 0.073	$z =$ 0.31
	**Sample Size**	UV vs SV	$p =$ 0.945	$U =$ 19.00
	UV $n =$ 10		$r =$ 0.032	$z =$ 0.14
	SV $n =$ 9	UD vs SD	$p =$ 0.796	$U =$ 44.00
	UD $n =$ 4		$r =$ 0.086	$z =$ 0.31
	SD $n =$ 9	UV vs SD	$p =$ 0.412	$U =$ 61.00
			$r =$ 0.201	$z =$ 0.87
**Figure**	**Shapiro-Wilk test**	**Two-Way ANOVA**		
**S4B**	UV $p =$ 0.052			
AI Count	SV $p =$ 0.653	Interaction $F(3,26) =$ 0.096	, $p =$ 0.759 ,	partial $\eta^2 =$ 0.004
40W	UD $p =$ 0.797	Stress $F(1,26) =$ 6.834	, $p =$ 0.015 ,	partial $\eta^2 =$ 0.208
	SD $p =$ 0.417	Pregnancy $F(1,26) =$ 0.018	, $p =$ 0.894 ,	partial $\eta^2 =$ 0.001
	**Levene test**			
	$p =$ 0.178	post hoc (Bonferroni)		
	**Sample Size**	UV vs UD	$p =$ 0.710	
	UV $n =$ 10	SV vs SD	$p =$ 0.914	
	SV $n =$ 4	UV vs SV	$p =$ 0.066	
	UD $n =$ 9	UD vs SD	$p =$ 0.087	
	SD $n =$ 7	UV vs SD	$p =$ 0.237	
**Figure**	**Shapiro-Wilk test**	**Two-Way ANOVA**		
**S4B**	UV $p =$ 0.562			
AI Area	SV $p =$ 0.304	Interaction $F(3,26) =$ 2.127	, $p =$ 0.157 ,	partial $\eta^2 =$ 0.076
40W	UD $p =$ 0.544	Stress $F(1,26) =$ 4.382	, $p =$ 0.046 ,	partial $\eta^2 =$ 0.144
	SD $p =$ 0.265	Pregnancy $F(1,26) =$ 0.355	, $p =$ 0.557 ,	partial $\eta^2 =$ 0.013

	**Levene test**	
	p = 0.646	post hoc (Bonferroni)
	**Sample Size**	UV vs UD *p* = 0.094
	UV *n* = 10	SV vs SD *p* = 0.597
	SV *n* = 4	UV vs SV *p* = 0.028
	UD *n* = 9	UD vs SD *p* = 0.629
	SD *n* = 7	UV vs SD *p* = 0.262
**Figure S4B**	**Shapiro-Wilk test**	**Mann Whitney U test**
	UV *p* = 0.288	UV vs UD *p* = 0.008 *U* = 58.00
AI Size	SV *p* = 0.260	*r* = 0.599 *z* = 2.61
40W	UD *p* = 0.017	SV vs SD *p* = 0.412 *U* = 19.00
	SD *p* = 0.872	*r* = 0.285 *z* = 0.95
	**Sample Size**	UV vs SV *p* = 0.304 *U* = 12.00
	UV *n* = 10	*r* = 0.302 *z* = 1.13
	SV *n* = 4	UD vs SD *p* = 0.071 *U* = 49.00
	UD *n* = 9	*r* = 0.463 *z* = 1.85
	SD *n* = 7	UV vs SD *p* = 0.669 *U* = 30.00
		r = 0.118 *z* = 0.49
**Figure S4C**	**Shapiro-Wilk test**	**Two-Way ANOVA**
	UV *p* = 0.739	
PrL Count	SV *p* = 0.900	Interaction F(3,26) = 1.138 , *p* = 0.296 , partial η^2 = 0.042
40W	UD *p* = 0.218	Stress F(1,26) = 0.388 , *p* = 0.539 , partial η^2 = 0.015
	SD *p* = 0.331	Pregnancy F(1,26) = 0.023 , *p* = 0.881 , partial η^2 = 0.001
	**Levene test**	
	p = 0.832	post hoc (Bonferroni)
	**Sample Size**	UV vs UD *p* = 0.446
	UV *n* = 10	SV vs SD *p* = 0.457
	SV *n* = 4	UV vs SV *p* = 0.773
	UD *n* = 9	UD vs SD *p* = 0.204
	SD *n* = 7	UV vs SD *p* = 1.000
**Figure S4C**	**Shapiro-Wilk test**	**Two-Way ANOVA**
	UV *p* = 0.898	
PrL Area	SV *p* = 0.453	Interaction F(3,26) = 1.363 , *p* = 0.254 , partial η^2 = 0.050
40W	UD *p* = 0.403	Stress F(1,26) = 0.506 , *p* = 0.483 , partial η^2 = 0.019
	SD *p* = 0.144	Pregnancy F(1,26) = 0.047 , *p* = 0.830 , partial η^2 = 0.002
	**Levene test**	
	p = 0.264	post hoc (Bonferroni)
	**Sample Size**	UV vs UD *p* = 0.252
	UV *n* = 10	SV vs SD *p* = 0.561
	SV *n* = 4	UV vs SV *p* = 0.228
	UD *n* = 9	UD vs SD *p* = 0.728
	SD *n* = 7	UV vs SD *p* = 1.000
**Figure S4C**	**Shapiro-Wilk test**	**Two-Way ANOVA**
	UV *p* = 0.741	
PrL Size	SV *p* = 0.877	Interaction F(3,26) = 0.016 , *p* = 0.899 , partial η^2 = 0.001
40W	UD *p* = 0.085	Stress F(1,26) = 2.226 , *p* = 0.148 , partial η^2 = 0.079
	SD *p* = 0.999	Pregnancy F(1,26) = 0.202 , *p* = 0.657 , partial η^2 = 0.008
	**Levene test**	

	p = 0.841	post hoc (Bonferroni)
	**Sample Size**	UV vs UD p = 0.629
	UV n = 10	SV vs SD p = 0.844
	SV n = 4	UV vs SV p = 0.297
	UD n = 9	UD vs SD p = 0.303
	SD n = 7	UV vs SD p = 0.827
**Figure**	**Shapiro-Wilk test**	**Mann Whitney U test**
**S4D**	UV p = 0.008	UV vs UD p = 0.145 U = 14.50
DG Count	SV p = 0.210	r = 0.380 z = 1.47
40W	UD p = 0.654	SV vs SD p = 0.315 U = 8.00
	SD p = 0.093	r = 0.341 z = 1.13
	**Sample Size**	UV vs SV p = 0.352 U = 17.00
	UV n = 6	r = 0.335 z = 1.06
	SV n = 4	UD vs SD p = 0.408 U = 23.00
	UD n = 9	r = 0.225 z = 0.90
	SD n = 7	UV vs SD p = 0.035 U = 6.00
		r = 0.594 z = 2.14
**Figure**	**Shapiro-Wilk test**	**Mann Whitney U test**
**S4D**	UV p = 0.036	UV vs UD p = 0.224 U = 15.00
DG Area	SV p = 0.707	r = 0.335 z = 1.30
40W	UD p = 0.595	SV vs SD p = 0.315 U = 8.00
	SD p = 0.066	r = 0.342 z = 1.13
	**Sample Size**	UV vs SV p = 0.010 U = 0.00
	UV n = 6	r = 0.809 z = 2.56
	SV n = 4	UD vs SD p = 0.837 U = 29.00
	UD n = 9	r = 0.066 z = 0.27
	SD n = 7	UV vs SD p = 0.035 U = 6.50
		r = 0.574 z = 2.07
**Figure**	**Shapiro-Wilk test**	**Mann Whitney U test**
**S4D**	UV p = 0.056	UV vs UD p = 0.864 U = 25.00
DG Size	SV p = 0.450	r = 0.061 z = 0.24
40W	UD p = 0.218	SV vs SD p = 1.000 U = 14.00
	SD p = 0.457	r = 0.000 z = 0.00
	**Sample Size**	UV vs SV p = 0.352 U = 7.00
	UV n = 6	r = 0.335 z = 1.06
	SV n = 4	UD vs SD p = 0.958 U = 32.00
	UD n = 9	r = 0.013 z = 0.05
	SD n = 7	UV vs SD p = 0.366 U = 14.00
		r = 0.277 z = 1.00
**Figure**	**Shapiro-Wilk test**	**Two-Way ANOVA**
**S5A**	UV p = 0.526	
AI Area	SV p = 0.232	Interaction F(3,33) = 2.875 , p = 0.099 , partial η^2 = 0.080
16W	UD p = 0.884	Stress F(1,33) = 0.221 , p = 0.641 , partial η^2 = 0.007
	SD p = 0.390	Pregnancy F(1,33) = 0.141 , p = 0.710 , partial η^2 = 0.004
	**Levene test**	
	p = 0.245	post hoc (Bonferroni)
	**Sample Size**	UV vs UD p = 0.333
	UV n = 10	SV vs SD p = 0.171
	SV n = 10	UV vs SV p = 0.117
	UD n = 10	UD vs SD p = 0.414
	SD n = 7	UV vs SD p = 0.487

**Figure**	**Shapiro-Wilk test**	**Two-Way ANOVA**
**S5A**	UV *p* = 0.768	
AI Size	SV *p* = 0.842	Interaction F(3,33) = 0.495 , *p* = 0.487 , partial η^2 = 0.015
16W	UD *p* = 0.086	Stress F(1,33) = 0.240 , *p* = 0.627 , partial η^2 = 0.007
	SD *p* = 0.492	Pregnancy F(1,33) = 0.472 , *p* = 0.497 , partial η^2 = 0.014
	**Levene test**	
	p = 0.580	post hoc (Bonferroni)
	**Sample Size**	UV vs UD *p* = 0.990
	UV *n* = 10	SV vs SD *p* = 0.355
	SV *n* = 10	UV vs SV *p* = 0.381
	UD *n* = 10	UD vs SD *p* = 0.886
	SD *n* = 7	UV vs SD *p* = 0.889
**Figure**	**Shapiro-Wilk test**	**Two-Way ANOVA**
**S5B**	UV *p* = 0.604	
PrL Area	SV *p* = 0.737	Interaction F(3,29) = 1.986 , *p* = 0.169 , partial η^2 = 0.064
16W	UD *p* = 0.807	Stress F(1,29) = 0.160 , *p* = 0.692 , partial η^2 = 0.005
	SD *p* = 0.178	Pregnancy F(1,29) = 0.104 , *p* = 0.776 , partial η^2 = 0.003
	**Levene test**	
	p = 0.165	post hoc (Bonferroni)
	**Sample Size**	UV vs UD *p* = 0.408
	UV *n* = 10	SV vs SD *p* = 0.709
	SV *n* = 8	UV vs SV *p* = 0.215
	UD *n* = 9	UD vs SD *p* = 0.937
	SD *n* = 10	UV vs SD *p* = 0.941
**Figure**	**Shapiro-Wilk test**	**Mann Whitney U test**
**S5B**	UV *p* = 0.925	UV vs UD *p* = 0.315 *U* = 24.00
PrL Size	SV *p* = 0.327	*r* = 0.260 *z* = 1.07
16W	UD *p* = 0.388	SV vs SD *p* = 0.536 *U* = 25.00
	SD *p* = 0.025	*r* = 0.172 *z* = 0.69
	**Sample Size**	UV vs SV *p* = 0.813 *U* = 32.00
	UV *n* = 10	*r* = 0.071 *z* = 0.29
	SV *n* = 7	UD vs SD *p* = 1.000 *U* = 32.00
	UD *n* = 7	*r* = 0.013 *z* = 0.05
	SD *n* = 9	UV vs SD *p* = 0.315 *U* = 32.00
		r = 0.243 *z* = 1.06
**Figure**	**Shapiro-Wilk test**	**Mann Whitney U test**
**S5C**	UV *p* = 0.626	UV vs UD *p* = 0.436 *U* = 39.00
DG Area	SV *p* = 0.053	*r* = 0.196 *z* = 0.83
16W	UD *p* = 0.009	SV vs SD *p* = 0.027 *U* = 15.00
	SD *p* = 0.094	*r* = 0.497 *z* = 2.22
	**Sample Size**	UV vs SV *p* = 0.739 *U* = 55.00
	UV *n* = 10	*r* = 0.085 *z* = 0.38
	SV *n* = 10	UD vs SD *p* = 0.696 *U* = 35.00
	UD *n* = 8	*r* = 0.105 *z* = 0.44
	SD *n* = 10	UV vs SD *p* = 0.156 *U* = 27.00
		r = 0.329 *z* = 1.47
**Figure**	**Shapiro-Wilk test**	**Mann Whitney U test**
**S5C**	UV *p* = 0.076	UV vs UD *p* = 0.579 *U* = 58.00

DG Size 16W	SV p = 0.012		r = 0.143	z = 0.61
	UD p = 0.037	SV vs SD	p = 0.021	U = 14.00
	SD p = 0.235		r = 0.517	z = 2.31
	**Sample Size**	UV vs SV	p = 0.813	U = 32.00
	UV n = 10		r = 0.066	z = 0.29
	SV n = 10	UD vs SD	p = 0.762	U = 36.00
	UD n = 8		r = 0.084	z = 0.36
	SD n = 10	UV vs SD	p = 0.211	U = 29.00
			r = 0.291	z = 1.30
**Figure S6A** AI NORT 16W	**Linear Regression and Pearson's Correlation**	$F(1,25)$ = 0.114, p = 0.738	R^2 = 0.004	
**Figure S6A** PrL NORT 16W	**Linear Regression and Pearson's Correlation**	$F(1,23)$ = 0.051, p = 0.822	R^2 = 0.002	
**Figure S6A** DG NORT 16W	**Linear Regression and Pearson's Correlation**	$F(1,27)$ = 1.108, p = 0.301	R^2 = 0.039	
**Figure S6B** AI S-trial 16W	**Linear Regression and Pearson's Correlation**	$F(1,34)$ = 0.715, p = 0.403	R^2 = 0.020	
**Figure S6B** PrL S-trial 16W	**Linear Regression and Pearson's Correlation**	$F(1,31)$ = 0.036, p = 0.849	R^2 = 0.001	
**Figure S6B** DG S-trial 16W	**Linear Regression and Pearson's Correlation**	$F(1,36)$ = 0.031, p = 0.861	R^2 < 0.001	
**Figure S6C** AI SN-trial 16W	**Linear Regression and Pearson's Correlation**	$F(1,34)$ = 0.856, p = 0.361	R^2 = 0.024	
**Figure S6C** PrL SN-trial 16W	**Linear Regression and Pearson's Correlation**	$F(1,31)$ = 0.918, p = 0.345	R^2 = 0.028	
**Figure S6C** DG SN-trial 16W	**Linear Regression and Pearson's Correlation**	$F(1,36)$ = 0.396, p = 0.532	R^2 = 0.010	
